# Local Reference Ranges of Thyroid Volume in Sudanese Normal Subjects Using Ultrasound

**DOI:** 10.4061/2011/935141

**Published:** 2011-09-26

**Authors:** Mohamed Yousef, Abdelmoneim Sulieman, Bushra Ahmed, Alsafi Abdella, Khaled Eltom

**Affiliations:** ^1^College of Medical Radiologic Science, Sudan University of Science and Technology, Baladya Street, P.O. Box 1908, Khartoum 11111, Sudan; ^2^College of Radiology and Nuclear Medicine, The National Ribat University, Nile Street, Burri, P.O. Box 55, Khartoum 11111, Sudan; ^3^Radiation and Isotope Center, Khartoum (RICK), Algaser Street, P.O. Box 846, Khartoum 11111, Sudan

## Abstract

This study aimed to establish a local reference of thyroid volume in Sudanese normal subjects using ultrasound. A total of 103 healthy subjects were studied, 28 (27.18%) females and 75 (72.82%) males. Thyroid volume was estimated using *ellipsoid formula*. The mean age and range of the subjects was 21.8 (19–29) years; the mean body mass index (BMI) was 22.3 (16.46–26.07) kg/m^2^. The overall mean volume ± SD volume of the thyroid gland for both lobes in all the patients studied was 6.44 ± 2.44 mL. The mean volume for both lobes in females and males were 5.78 ± 1.96 mL and 6.69 ± 2.56 mL, respectively. The males' thyroid volume was greater than the females'. The mean volume of the right and left lobes of the thyroid gland in males and females were 3.38 ± 1.37 mL and 3.09 ± 1.24 mL, respectively. The right thyroid lobe volume was greater than the left. The values obtained in this study were lower than those reported from previous studies.

## 1. Introduction

Ultrasound has become one of the primary imaging modalities for the assessment of the major glands of internal secretion within the cervical region. The thyroid gland is among the most commonly imaged glands using ultrasound due to the limitation of clinical examination [1]. Computed tomography (CT) and magnetic resonance imaging (MRI) provide structural information of the thyroid gland just like ultrasound but are relatively more expensive. Thyroid ultrasound appears suitable in tropical Africa [2, 3] where more sophisticated modern imaging techniques may not be readily available or are very expensive. 

Anatomically, the normal thyroid gland consists of two lobes which lie on the anterolateral surface of the trachea extending from the thyroid cartilage superiorly to the sixth tracheal ring inferiorly. They are asymmetrical with the right lobe being larger than the left, and the thyroid gland is larger in males [4, 5]. In recent decades, sonography has become the gold standard for assessment of the thyroid gland [6].

Sonography has improved with the development of high-frequency transducers, which allow a more detailed study of the thyroid gland [7]. As a result, the World Health Organization (WHO) and the International Council for the Control of Iodine Deficiency Disorders (ICCIDD) now consider sonography the diagnostic method for assessment of goiter [8]. It is most often used in assessing the incidence of goiter in Third World populations, especially in children [9]. Intra- and interobserver variation can lead to differences in volume calculation, irrespective of the correction factor. Nevertheless, a more optimal correction factor will give a more realistic measurement of thyroid volume.

Volumetric evaluation of the thyroid gland is based on the use of an ellipsoid model. Hence, a value is obtained that replaces clinical evaluation of volume. With the ellipsoid model, the height, the width, and the depth of each lobe are measured and multiplied. The obtained result is then multiplied by a correction factor [10].

The work of Brunn et al. [11] in 1981 was based on volume measurement of cadaver glands subsequently immersed in water.

Brunn et al. [11] concluded that a modified correction factor of 0.479 resulted in a more accurate assessment of thyroid volume compared with the previously accepted correction factor of *π*/6 or 0.524.

In Sudan, there is absence of domestic reference for thyroid volumes; in Sudan, as for as we know, no study was published in the open literature, regarding the thyroid volume. 

This study aimed to establish a local reference of thyroid volume in Sudanese normal subjects using ultrasound.

## 2. Materials and Methods

This study was done in the Sudan University of Science and Technology, College of Medical Radiological Science during the period from 2007 up to 2010. 

### 2.1. Ultrasound Machines

The ultrasound system used is general electric (GE) medical system, made by Yokogawa medical system, Ltd., 7-127 Asahigaoka 4-chome, Hino-shi Tokyo, Japan. Model 2302650 with serial of 1028924YM7 and manufacturing date of April 2005, a grey scale real-time ultrasound machine, fitted with a 10 MHz transducer was used for the study. 

### 2.2. Volunteers

A total of 103 healthy students from the Sudan University of Science and Technology, College of Medical Radiologic Sciences were involved in this study. The ethics and research committee approved the study, and consents were obtained from all volunteers prior to the examination. 

### 2.3. Exclusion Criteria

Subjects with anterior neck swelling or clinical evidence of thyroid disease were excluded. Furthermore, women during menstruation, pregnant, women who have delivered within the last 12 months, were excluded from the study because this may affect the thyroid size. The data was collected and analyzed using SPSS for windows version 17. 

### 2.4. Measurement Technique for Thyroid Volume

With the ellipsoid model, the height, the width, and the depth of each lobe are measured and multiplied. The obtained result was then multiplied by a correction factor, which is *π*/6 or 0.524 [12]. The subjects were examined in supine position, with pillow placed under their shoulders to hyperextend the neck. US gel was applied over the thyroid area. The transducer was directly placed on the skin over the thyroid gland, and an image of each lobe was obtained in transverse and longitudinal planes. The craniocaudal and the sagittal dimensions of both lobes were measured on the longitudinal image. The transverse dimension was measured on the transverse image.

## 3. Results

**Figure 1:**
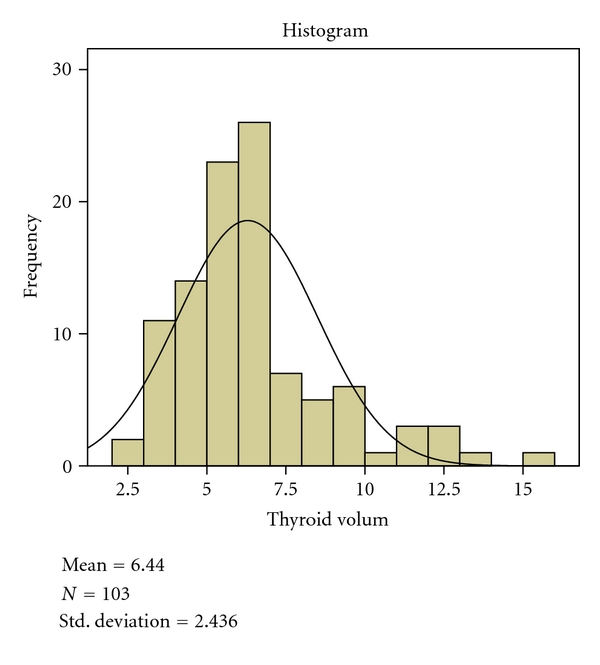


The 103 subjects studied consist of 28 (27.18 %) females and 75 (72.82%) males. The mean age of the subjects was 21.79 years with a range of 19–29 years. The overall mean volume of the thyroid gland for both lobes in all the patients studied was 6.44 ± 2.44 (Table 1 and Figure 1). The mean volume for both lobes in females and males was 5.78 ± 2 (1.96) mL and 6.69 (2.56)  mL, respectively. The mean volume of the right and left lobes of the thyroid gland in all the patients studied were 3.38 ± 2 (1.37) mL and 3.09 ± 2 (1.24) mL, respectively (Table 1). The right thyroid lobe volume was greater than the left.

The mean thyroid volume of the right lobe among the females studied was 3.03 mL, and the left was 2.75 mL (Table 1). The values were greater for the right than the left lobe. In males, the right and the left lobes of the thyroid gland volumes were 3.51 mL and 3.21 mL, respectively, (Table 1). The values were greater for the right than the left lobe and more than that of the females.

## 4. Discussion

In recent decades, the WHO has changed the diagnostic criteria for goiter. The diagnosis of goiter used to be based on palpation, but now it is based on volume measurement using sonography. Volume measurement of the thyroid gland is especially easy to obtain because the gland has a different echogenicity compared with adjacent soft tissues [11]. Due to its conical morphology, a thyroid lobe is assumed to resemble an ellipsoid, and its volume is approximated using height × width × depth × a correction factor. Other methods such as the 3D sonography and the automated transverse surface area method have been proposed to evaluate thyroid volume [13, 14].

Thyroid lobes, however, show variations in shape as is evident in anatomic and imaging studies [15, 16]. Failure of the thyroid gland to descend from foramen caecum along the thyroglossal duct to the anterior aspect of the neck accounts for the rare ectopic location of the thyroid tissue at the base of the tongue (lingual thyroid) as well as the presence of thyroglossal duct cyst along this developmental tract [12]. The thyroid size was found to increase during pregnancy and decreases up to 12 months postpartum period [17, 18]. The menstrual cycle also seems to associate with cyclical alteration of thyroid size in healthy women [19], and, for that reasons, these subjects were excluded from this study.

The overall mean thyroid gland volume combined for both lobes and sexes obtained from this study was 6.44 cm^3^. There was no previous local study for comparison to the best of our knowledge. But in Africa, Anele [3] studied the thyroid gland volume among Nigerians. This value showed the thyroid dimensions to be slightly lower than the Western values [5, 20].

This study has shown that the right thyroid lobe volume (3.38 mL) was greater than the left (3.09 mL) with significant statistical difference between the right and the left lobe volumes in both sexes. This finding is in agreement with previous studies done among the Caucasians and the Chinese [5, 20, 21].

The total mean values for the females (5.78 mL) and the males (6.69 mL) have shown the thyroid gland to be greater in males compared to females. Anele [3] found no significant difference in the thyroid volume between males and females. This finding differs from our study and most of the previous studies [5, 20–22]. 

In conclusion, the thyroid volume obtained in this study was in the lower range of the values reported in previous studies (Table 2). The volume of the right lobe of the gland was greater than the left in both sexes. The mean thyroid volume in the males is greater than that in the females, a local reference of thyroid volume was established, and further studies are required to establish national references thyroid volume in Sudan. 

## Figures and Tables

**Table 1:** Volume of the thyroid gland.

Gender	Thyroid volume	Right lobe volume	Left lobe volume
Female			
Mean	5.78	3.03	2.75
*N*	28	28	28
Std. deviation	1.96	1.02	1.05
Male			
Mean	6.69	3.51	3.21
*N*	75	75	75
Std. deviation	2.56	1.46	1.28
Total			
Mean	6.44	3.38	3.09
*N*	103	103	103
Std. deviation	2.44	1.37	1.24

**Table 2:** Comparison of thyroid volume studies.

Author	Gender	Age range (years)	Number of subjects	Thyroid volume (mL) ± SD	Country
Current study	75 M 28 F	19–29	103	6.44 ± 2.44	Sudan
Ivanac et al. [23]		20–38	51	10.68 ± 2.83	Croatia
Ahidjo et al. [24]	71 M 72 F	23–69	143	8.55 ± 1.82	Nigeria
Chanoine et al. [25]		17–20	256	11.6 ± 4.4	Belgium
Adibi et al. [26]	123 M 77 F	37.27 ± 11.80	200	9.53 ± 3.68	Iran
